# Long COVID Symptoms vs. Back Pain and Physical Activity among Students in Poland—Cross-Sectional Study

**DOI:** 10.3390/jcm13041038

**Published:** 2024-02-11

**Authors:** Monika Gałczyk, Anna Zalewska

**Affiliations:** Faculty of Health Sciences, University of Lomza, 14 Akademicka St., 18-400 Lomza, Poland; aanna.zalewska@gmail.com

**Keywords:** long COVID, students, physical activity, neck pain, low back pain

## Abstract

Background: Back pain (BP) is a common condition that affects people of all ages. Moderate- and vigorous-intensity physical activity (PA) is a key element in maintaining health. The purpose of this research was to determine the level of physical activity and back pain (BP) in students with long COVID symptoms and to determine the relationship between the level of PA and BP in students with and without long COVID. Methods: A survey was carried out among 402 students from Poland. The inclusion criteria were as follows: student status, age of over 18 years, history of COVID-19, and consent to participate in the study. The International Physical Activity Questionnaire (IPAQ) was used to determine the level of PA. The Oswestry Disability Index (ODI) and the Neck Disability Index (NDI) were used to assess BP. Results: We found that at least half of the students surveyed did not experience any lumbosacral or cervical spine pain. The authors found no association between the level of PA in women and a history of long COVID symptoms, while there were statistically significant differences in intense PA in men (*p* = 0.0263), with those who did not report long COVID symptoms being more active. With regard to cervical and lumbar spine pain complaints, in our study, these were statistically significantly stronger in students who were observed to have long COVID symptoms. The difference was not significant only for lumbosacral complaints among men. No strong correlations were found between PA level and the severity of BP. Conclusion: Additional investigation is required to comprehend the complex interaction between long COVID symptoms and levels of PA and BP. Special attention should be paid to the prevention of back pain mainly in the COVID-19 group of students.

## 1. Introduction

In 2020, the world faced a unique challenge in the form of a COVID-19 pandemic brought on by the SARS-CoV-2 virus. Step by step, scientists tried to understand the virus and develop effective treatment and prevention strategies [1,2,3,4]. Over time, patients gradually recovered from COVID-19, but more and more questions arose about their health and quality of life after recovery. There are reports from all over the world of patients who, after testing positive for COVID-19 and overcoming the acute phase of the disease, do not return to their normal daily activities because they still have some of the symptoms of the disease that persist for more than a month [5,6,7]. This group of patients, which accounts for between 10% and 20%, reports multiple and complex symptoms such as fatigue, post-exertional malaise, shortness of breath, headaches, and many others that affect the ability to perform daily activities [8,9,10]. This condition has been defined as prolonged COVID-19 syndrome and has affected an increasing number of people as the pandemic has progressed [11,12,13,14]. According to the World Health Organization, more than 772 million people worldwide had contracted COVID-19, with almost 7 million deaths [15]. The vast majority of people have recovered, but the long-term consequences of the disease are not yet fully known. To this end, studies and observations are being conducted in many medical centers around the world on patients who have survived the new virus infection [16,17,18].

Moderate- and vigorous-intensity physical activity (PA) is a key element in maintaining health, including mental health, good physical fitness, normal body weight, and, ultimately, well-being [19,20,21,22]. PA is a general term that refers to any form of movement or PA that engages the body and its systems, including the muscles, heart, lungs, and bones. This can include a variety of activities such as exercise, sports, walking, gardening, housework, dancing, and many others. The first and most important benefit of regular PA is the reduction in the risk of many diseases. A previous survey has shown that PA has a positive effect on the human condition and that a lack of PA has a negative effect on the body [23,24,25,26,27,28].

Scientific reports indicate that people’s PA has decreased significantly during the pandemic, while exercise levels protect against the severe consequences of COVID-19 [29,30]. Healthy individuals are advised to maintain their current level of PA, as low PA leads to decreased fitness, muscular endurance, and increased insulin resistance [29,30].

BP is a common condition that affects people of all ages. It is estimated to affect 30% to 80% of the population. Most people with this problem tend to have recurring episodes of pain. BP syndrome can be related to work, frequent physical exertion, or obesity. Risk factors include obesity, lack of work ergonomics, type of work performed, and length of service [31].

The currently available studies show that, in survivors of virus infection, the occurrence of pain is mainly observed in the osteo-musculoskeletal system [32,33]. Reduced PA at this time, remote working, or weight gain may have contributed to a higher incidence of musculoskeletal pain [34].

A study conducted among students and employees of higher education institutions in Lodz found that the incidence and severity of musculoskeletal complaints increased among the respondents during the epidemic period. A greater severity of the complaints in question was noted with an increase in the proportion of working/learning time in remote form [35]. Another study in a group of teachers and university lecturers in Poland found a correlation between the onset of BP and the shift in the nature of work following the COVID-19 pandemic announcement. The average degree of reported cervical and lumbar back pain increased with the number of hours spent at a computer, and there was a statistically significant increase in pain intensity since the introduction of distance learning [36].

As the reports in the literature today show, long COVID syndrome is a significant public health problem worldwide. To shed more light on this topic, the authors of this article decided to conduct a study on a group of students who had contracted COVID-19. Their aim was to determine the level of PA and BP in students with long COVID symptoms and to determine the relationship between the level of PA and BP in students with and without long COVID.

## 2. Materials and Methods

### 2.1. Definitions

A diagnosis of COVID-19 in the following study means that the subjects had contracted COVID-19 at least once during the pandemic, which was confirmed by a positive result: a genetic test from nasopharyngeal swab material, or an antigen test from nasopharyngeal swab material, or a venous blood test for antibodies [37].

Long COVID syndrome in the following study means that participants observed a range of post-viral symptoms that lasted longer than 4 weeks and were not associated with a specific medical diagnosis. These were, among others, general symptoms (tiredness or fatigue that interferes with daily life, symptoms that get worse after physical or mental effort, and fever); respiratory and heart symptoms (difficulty breathing or shortness of breath, cough, chest pain, and fast-beating or pounding heart); neurological symptoms (difficulty thinking or concentrating headache, sleep problems, dizziness when you stand up, pins-and-needles feelings, change in smell or taste, and depression or anxiety); digestive symptoms (diarrhea and stomach pain); and other symptoms (joint or muscle pain, rash, and changes in menstrual cycles) [38].

Back pain in the following study is a term that describes neck pain and low back pain.

### 2.2. Participants and Procedure

The authors conducted a cross-sectional online survey of a group of Polish university students with COVID-19 from November to December 2023. The survey (Supplementary Materials) was distributed on e-learning platforms and via social media. The survey was anonymous and voluntary, and students could unsubscribe at any time. The survey also included information about the purpose of the research being conducted, as well as the definition of long COVID syndrome [38]. Inclusion criteria were as follows: student status, being over the age of majority, history of COVID-19, and consent to participate in the study. However, the presence of chronic musculoskeletal, cardiovascular, and respiratory diseases, as well as musculoskeletal injuries, excluded participation in the study. A total of 500 students participated in the study, of which 98 questionnaires were not completed completely or the subjects did not meet the inclusion criteria. A final total of 402 students were eligible for the study. The authors received approval from the Senate Committee on Ethics in Scientific Research of the University of Medical Sciences in Bialystok (KB/18/2020.2021).

Due to the fact that several numerical measures were used in the study (ODI, NDI, 4 IPAQ measures), it was difficult to establish clear criteria for the desired accuracy of the results and, on this basis, determine the sample size. For this reason, it was decided to focus on a selection of the sample to accurately estimate the percentage of people who suffered from back pain, defined as the sum of ODI and NDI at a level above 5 points (this variable was used in logistic regression model). Assuming a confidence level of 95%, an expected incidence of back pain of 40% [39], and a confidence interval of ±5%, the required sample size is 369 people. We managed to obtain data from a larger number of people, which allows us to conclude that the frequency of back pain reported in the study was estimated with an accuracy of not less than ±5%.

### 2.3. Methods for Assessing Study Variables

In the survey questionnaire, the authors assessed the level of BP using the Oswestry Disability Index (ODI) questionnaire, which assesses the lumbar section, and the Neck Disability Index (NDI), which assesses the cervical section. These are scales used for people who struggle with spine pain on a daily basis. The maximum number of points a patient can obtain is 50, where 0–4—no disability and 35–50—extreme suffering.

Cronbach’s alpha values for Oswestry Disability Index and Neck Disability Index reported in the literature are >0.7 [40,41].

The level of PA was assessed using a shortened Polish version of the International Physical Activity Questionnaire (IPAQ), which was developed for people aged 16–69 years. It contains seven questions assessing all types of daily activity lasting at least 10 min continuously. They are expressed in MET-min/week (MET—metabolic equivalent of labour). Cronbach’s alpha values for IPAQ reported in the literature are >0.7 [42,43,44].

### 2.4. Statistical Methods

For statistical analysis, Statistica v. 13 software was used (TIBCO Software Inc., 2017, Palo Alto, CA, USA). In view of the high asymmetry of the distribution of the IPAQ, ODI, and NDI, statistical inference regarding PA and BP complaints was conducted using non-parametric methods. The significance of the difference in PA levels between the long COVID group and the other students was determined using the Mann–Whitney test. The non-parametric Spearman’s rank correlation coefficient was used to test the correlation between PA level and BP. The final element of the statistical analysis was the construction of a logistic regression model assessing factors such as long COVID symptoms, age, and measures of PA as influencing factors for the occurrence of BP in students. A significance level of *p* < 0.05 was established for all statistical analyses.

## 3. Results

### 3.1. Description of the Study Group

The study was conducted among 402 students who had COVID-19. The study group was 60.2% female and 39.8% male. The average age of the students was 21.5 years with a standard deviation of 1.2 years. Almost two-thirds of the students were 21 or 22 years old. Slightly less than half of the students (45.5%) who had a COVID-19 infection showed the presence of long COVID symptoms. The area of study undertaken by the respondents is presented in the table (Table 1).

### 3.2. Physical Activity Level and Back Pain—Analysis for the Whole Population

The following table (Table 2) presents information on the level of PA and the level of BP. The summary includes a division according to sex. In addition, there is a certain overrepresentation of women in the study population, which makes it all the more necessary to take the sex factor into account in any analysis. Information on the distribution of the IPAQ and ODI and NDI measures includes the mean and standard deviation, median, lower and upper quartiles, and the value of the skewness coefficient.

Based on the values of the skewness coefficients and the comparison of medians and mean values, it can be concluded that the distribution of the IPAQ, ODI, and NDI measures shows a very pronounced right-sided asymmetry; i.e., low and medium values dominate, while there are only a few high values. This is particularly evident for the ODI and NDI measures, for which the median is 0 points (except for the NDI for men), meaning that at least half of the students do not experience any lumbosacral or cervical BP.

### 3.3. Long COVID Symptoms vs. Physical Activity and Back Pain

We analyzed whether the level of PA depended on the presence of long COVID symptoms (Table 3), followed by the same analysis for the presence of pain (Table 4). The analysis was broken down by sex, and the mean, median, standard deviation, and both quartiles were tabulated. As can be seen, there is no correlation between the level of PA in women and the occurrence of long COVID symptoms, while statistically significant differences are found in men with intense PA (*p* = 0.0263 *), with the activity being higher in those who do not report long COVID symptoms (median 720 vs. 560). The difference in walking is also almost statistically significant (*p* = 0.0610), and the median is higher in the group of people who reported themselves with long COVID symptoms (396 vs. 330).

As far as BP complaints are concerned, they are statistically significantly stronger among those declaring long COVID symptoms. The difference is only not significant for lumbosacral complaints among men.

**Figure 1:**
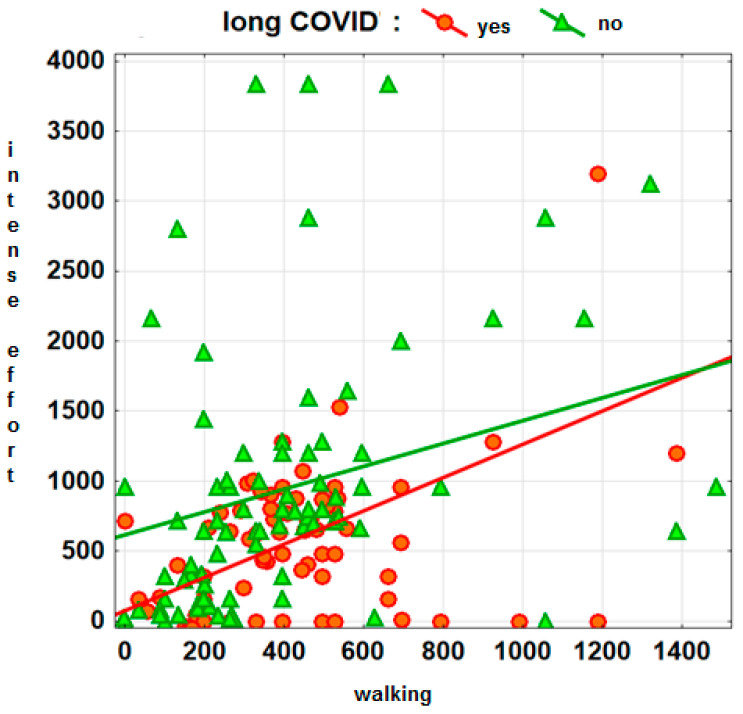
Long COVID symptoms and PA in men.

An illustration of the results of the above analysis can be found in the graph (Figure 1), which clearly shows that men reporting no prolonged COVID symptoms have higher levels of vigorous activity and slightly lower levels of walking—simple regressions are helpful in interpreting the graph to identify areas of marker concentration corresponding to individuals in both comparison groups.

### 3.4. Correlations between Activity Level and Pain Complaints

A correlation analysis was conducted between the level of PA (according to IPAQ measures) and the severity of BP (Table 5). The analysis was broken down by the sex of the students.

No strong correlations were found, with the only statistically significant correlation being between the severity of cervical spine pain and moderate-intensity activity level (*r*_S_ = −0.17; *p* = 0.0324 *).

An additional analysis was carried out, taking into account the occurrence of complaints described by the respondents as long COVID (Table 6). However, even the inclusion of this additional control factor does not appear to lead to a correlation between BP and PA. In the group with long COVID, there is no statistically significant correlation, while, in the group without long COVID, there are two statistically significant correlations, but their strength is very low.

BP incidence was defined by the sum of the ODI and NDI values. Individuals for whom the sum of the ODI and NDI scores exceeded 5 points were filtered out. There were 168 such individuals in the total study population, corresponding to approximately 42% of the total study population (44% and 38% in the female and male groups, respectively). The regression models were constructed separately for the group of women and men, assuming that gender determines the way in which the factors influencing blood pressure risk interact. The optimal model, containing only statistically significant variables, was sought using a stepwise regression procedure. The results obtained are presented in Table 7.

For the male group, the only significant factor found to increase BP risk was the presence of long COVID symptoms (odds ratio 2.356). The presence of long COVID symptoms also increased the risk of BP in the female group, and to a similar extent (OR = 2.269). In women, physical activity also had a significant effect on the occurrence of blood pressure, with intense physical activity increasing the risk of blood pressure and moderate physical activity decreasing it (OR = 1.178 and OR = 0.229).

## 4. Discussion

In our research, there is no correlation between the level of PA in women and the occurrence of long COVID symptoms, while statistically significant differences are found in men with intense PA, with the activity being higher in those who do not report long COVID symptoms. As far as BP complaints are concerned, they are statistically significantly stronger among those declaring long COVID symptoms. The difference is only not significant for lumbosacral complaints among men.

A meta-analysis by Papalia et al. in 2023 found a statistically significant increase in the severity (SMD −1.40, 95% CI −2.18 to −0.63, *p* = 0.0004) and prevalence (OR 0.53, 95% CI 0.29 to 0.96, *p* = 0.04) of lumbar BP in different population groups compared to the pre-pandemic and pandemic periods [45]. The conclusions regarding lumbar and cervical spine pain are supported by studies conducted among students worldwide during and after the pandemic [46,47].

There are reports in the literature in which no significant differences in the prevalence of BP were reported, e.g., among physiotherapy students in Israel [48]. In our study, we found that at least half of the students surveyed did not experience any lumbosacral or cervical spine pain.

The relationship between long COVID symptoms and PA levels is unclear [49]. COVID-19 patients show a significant reduction in walking time about 3 months after infection and a partial improvement in walking time 6 months after COVID [50]. The pattern of recovery in COVID-19 is compared with that established for influenza A [51,52]. Patients recovering from COVID-19 are encouraged to return to performing daily PA, and to begin low-/moderate-intensity exercise [30]. There are reports in the literature that there is no correlation between different durations of long COVID and PA levels [49]. In their study, the authors found no association between the level of PA in women and a history of long COVID symptoms, while there were statistically significant differences in intense PA in men (*p* = 0.0263), with those who did not report long COVID symptoms being more active. Bearing in mind that PA can contribute to both the improvement and worsening of some long COVID symptoms such as fatigue, for example, this may confirm the results [49,53]. A similar statistically significant (*p* = 0.0610) difference as in our study was also found for walking: the median is higher in the group of people with long COVID symptoms. However, this can be explained to some extent—people who feel worse (whether due to long COVID or other factors) are less likely to undertake intensive efforts, while they may be more likely to attempt lower-intensity activities. In addition, unknown confounding factors, such as the severity of COVID-19, length of observation of long COVID symptoms, or level of PA prior to the disease, could influence the results and play a role in this study.

Musculoskeletal pain is reported by patients as one of the most commonly observed symptoms of long COVID-19 syndrome, with the overall prevalence reported in the literature ranging from 0.3 per cent to 65.2 per cent, with the female sex appearing to be a potential risk factor [54,55]. With regard to cervical and lumbar spine pain complaints, in our study, these were statistically significantly stronger in students who were observed to have long COVID symptoms. The difference was not significant only for lumbosacral complaints among men.

No strong correlations were found between PA level and the severity of BP. The only statistically significant correlation was between the severity of cervical spine pain and moderate-intensity activity level (r_S_ = −0.17; *p* = 0.0324). However, this correlation was very weak and its practical significance is low. The decrease in moderate-intensity activity in men with more severe BP is small. In conclusion, there is no correlation between PA and the severity of BP. The results obtained are consistent with some reports in the literature [56]. The lack of correlation could be related to the fact that there were only a few students with severe pain in the study group and that mild pain, which also occurs in a minority of people, should not interfere with PA. Secondly, spinal problems may be a reason for additional PA in the context of rehabilitation recommendations. Third, PA is influenced by a number of other factors related not only to physical condition and health status, but also to volitional characteristics—many healthy people are not physically active enough, and vice versa [57,58].

Strategies to increase physical activity in patients with long COVID-19 are an important element to accelerate the recovery of SARS-CoV-2-infected patients. Rehabilitation with physical activity is a promising strategy used in routine clinical practice, although its therapeutic effects are not yet clearly defined. The literature shows that it has a positive effect on the symptoms of dyspnoea, fatigue, depression, and quality of life [59].

The study presented by the authors has several limitations that need to be considered. The most important is the cross-sectional nature of the study, as it does not provide clear evidence for the observed relationships. Another limitation is the use of social media for data collection, which means that people who do not use them were excluded. Additionally, all the data were collected using self-report questionnaires, which may affect the responses obtained. The authors did not collect the student’s vaccination status and the time elapsed between infection or long COVID symptoms and the last vaccination. Vaccination status may have influenced the history of long-term COVID and the magnitude of back pain.

The submitted manuscript also has a number of strengths. To the authors’ knowledge, this is the first study to examine complaints of BP and levels of PA in long COVID students. Future studies should include a larger sample and other populations and use more objective methods to assess PA and BP.

## 5. Conclusions

Further research is needed to understand the complex interaction between long COVID symptoms and levels of PA and BP. Special attention should be paid to the prevention of BP mainly in the long COVID-19 group of students.

## Figures and Tables

**Table 1:** Study area.

Study Area	Number	Percentage
Medical	90	22.4%
Economics	29	7.2%
Humanities	23	5.7%
Pedagogical	31	7.7%
Psychological	15	3.7%
Exact sciences	12	3.0%
Legal	30	7.5%
Administrative	65	16.2%
Artistic	14	3.5%
Biology and nature	10	2.5%
Philological	42	10.4%
Technical	25	6.2%
Tourism and sport	10	2.5%
Social	4	1.0%
Agricultural and forestry	1	0.2%
Military and naval	1	0.2%

**Table 2:** Information on level of PA and complaints of BP.

PA and BP	Sex											
	Woman (*N* = 242)						Man (*N* = 160)					
	Mean	Median	Std. Dev.	Lower Quartile	Upper Quartile	Skewness	Mean	Median	Std. Dev.	Lower Quartile	Upper Quartile	Skewness
PA (IPAQ)												
Intense effort	611	640	631	320	800	4.98	828	720	1 009	160	960	2.86
Moderate effort	392	360	337	240	480	3.71	464	360	454	240	480	3.18
Walking	423	330	372	264	396	3.82	463	396	487	215	495	4.86
Total effort	1426	1377	969	980	1704	3.93	1755	1451	1609	827	1935	2.58
BP												
ODI	2.93	0	4.96	0	5	2.38	2.35	0	4.84	0	3	2.86
NDI	4.21	2	5.42	0	6	2.17	3.54	2	4.43	0	6.5	1.31

PA—physical activity, BP—back pain, NDI—Neck Disability Index, ODI—Oswestry Disability Index, IPAQ—International Physical Activity Questionnaire.

**Table 3:** Long COVID symptoms and PA.

PA	Symptoms of Long COVID-19										*p*
	Yes					No					
	Mean	Me	*s*	*Q*_1_	*Q*_3_	Mean	Me	*s*	*Q*_1_	*Q*_3_	
women											
Intense effort	649	640	818	320	720	578	640	407	240	800	0.5315
Moderate effort	386	360	331	240	480	398	360	342	240	480	0.4003
Walking	477	330	448	264	495	376	297	286	264	396	0.1483
Total effort	1512	1323	1235	968	1700	1352	1377	657	984	1704	0.7795
men											
Intense effort	612	560	716	160	720	1000	720	1167	160	1200	0.0263 *
Moderate effort	460	360	496	240	480	467	480	420	240	600	0.4003
Walking	452	396	269	297	528	472	330	609	198	495	0.0610
Total effort	1523	1356	1297	977	1695	1940	1577	1807	692	2255	0.1616

PA—physical activity, *p*—test probability value calculated using the Mann–Whitney test, *p* < 0.05 (*).

**Table 4:** Long COVID symptoms vs. pain complaints.

BP	Symptoms of Long COVID-19										*p*
	Yes					No					
	Mean	Me	*s*	*Q*_1_	*Q*_3_	Mean	Me	*s*	*Q*_1_	*Q*_3_	
women											
ODI	4.10	0	6.07	0	8.5	1.92	0	3.45	0	3	0.0034 **
NDI	5.72	5	6.36	0	10	2.90	0	4.05	0	6	0.0001 ***
men											
ODI	2.99	0	5.68	0	5	1.84	0	4.02	0	2	0.2925
NDI	4.76	3	4.80	0	10	2.56	0	3.86	0	4	0.0007 ***

*p*—test probability values calculated using the Mann–Whitney test, NDI—Neck Disability Index, ODI—Oswestry Disability Index, BP—back pain, *p* < 0.01 (**), *p* < 0.001 (***).

**Table 5:** Correlations between PA level and pain complaints.

IPAQ	Sex			
	Woman		Man	
	ODI	NDI	ODI	NDI
Intense effort	−0.02 (*p* = 0.7124)	−0.11 (*p* = 0.0894)	0.14 (*p* = 0.0703)	−0.02 (*p* = 0.8065)
Moderate effort	−0.04 (*p* = 0.5239)	−0.07 (*p* = 0.2737)	−0.06 (*p* = 0.4461)	−0.17 (*p* = 0.0324 *)
Walking	0.01 (*p* = 0.9244)	0.03 (*p* = 0.6669)	0.09 (*p* = 0.2802)	0.10 (*p* = 0.2103)
Total effort	0.05 (*p* = 0.4031)	0.00 (*p* = 0.9398)	0.09 (*p* = 0.2425)	−0.06 (*p* = 0.4669)

IPAQ—International Physical Activity Questionnaire, NDI—Neck Disability Index, ODI—Oswestry Disability Index, *p* < 0.05 (*).

**Table 6:** Correlations between level of PA, pain, and long COVID.

IPAQ	Sex			
	Woman		Man	
	ODI	NDI	ODI	NDI
**People with symptoms of long COVID**				
Intense effort	0.02 (*p* = 0.8609)	−0.02 (*p* = 0.8618)	0.07 (*p* = 0.5761)	0.01 (*p* = 0.9095)
Moderate effort	0.03 (*p* = 0.7641)	0.00 (*p* = 0.9863)	0.00 (*p* = 0.9701)	−0.22 (*p* = 0.0656)
Walking	0.03 (*p* = 0.7263)	0.02 (*p* = 0.8199)	0.07 (*p* = 0.5369)	0.17 (*p* = 0.1573)
Total effort	0.15 (*p* = 0.1183)	0.12 (*p* = 0.2069)	0.05 (*p* = 0.6825)	−0.04 (*p* = 0.7238)
**People without symptoms of long COVID**				
Intense effort	−0.06 (*p* = 0.531)	−0.18 (*p* = 0.0382 *)	0.25 (*p* = 0.0175 *)	0.08 (*p* = 0.4696)
Moderate effort	−0.07 (*p* = 0.4047)	−0.11 (*p* = 0.2080)	−0.10 (*p* = 0.3380)	−0.13 (*p* = 0.2254)
Walking	−0.03 (*p* = 0.7124)	−0.01 (*p* = 0.9093)	0.06 (*p* = 0.5542)	−0.01 (*p* = 0.957)
Total effort	−0.02 (*p* = 0.7802)	−0.09 (*p* = 0.3242)	0.15 (*p* = 0.1647)	−0.01 (*p* = 0.9413)

IPAQ—International Physical Activity Questionnaire, NDI—Neck Disability Index, ODI—Oswestry Disability Index, *p* < 0.05 (*).

**Table 7:** Risk of back pain—results of logistic regression.

Independent Factors	Occurrence of BP	
	OR (95% c.i.)	*p*
Woman		
Occurrence of long COVID-19	2.269 (1.337–3.852)	0.0024 **
Intense effort ^(BC)^	1.178 (1.007–1.379)	0.0413 *
Moderate effort ^(BC)^	0.229 (0.056–0.944)	0.0413 *
Man		
Occurrence of long COVID-19	2.356 (1.227–4.522)	0.0100 *

OR—odds ratio with 95% confidence interval, BC—activity measures were Box–Cox transformed to reduce the asymmetry of their distribution BP—back pain, *p* < 0.05 (*), *p* < 0.01 (**), .c.i.—confidence interval.

## Data Availability

The data that support the findings of this study and the relevant questionnaires are available from the corresponding author, [M.G.], upon reasonable request.

## Supplementary Materials

The following supporting information can be downloaded at: https://www.mdpi.com/article/10.3390/jcm13041038/s1, The survey: Long COVID Symptoms versus Back Pain and Physical Activity among Students in Poland—cross sectional study.
