# Supplementary material for: Long COVID Symptoms vs. Back Pain and Physical Activity among Students in Poland—Cross-Sectional Study
Source: J Clin Med. 2024 Feb 11;13(4):1038. doi: 10.3390/jcm13041038 (PMC10889772; doi:10.3390/jcm13041038)
Supplement: Supplementary file 1 [file jcm-13-01038-s001.zip › jcm-2845815-supplementary.pdf]

Long COVID Symptoms vs. Back Pain and Physical Activity among Students in Poland—Cross-Sectional Study

Szanowni Państwo,

ten kwestionariusz jest przeznaczony dla studentów. Jego celem jest ocena poziomu aktywności fizycznej i dolegliwości bólowych kręgosłupa u studentów z objawami long COVID oraz określić związek pomiędzy poziomem aktywności fizycznej i dolegliwości bólowych kręgosłupa u studentów z objawami long COVID i bez nich. Uzyskane dane są całkowicie poufne i zanonimizowane na wszystkich etapach badania, będą wykorzystywane wyłącznie do celów naukowo-badawczych. Dane będą prezentowane i publikowane w sposób uniemożliwiający identyfikację respondentów.
Badanie realizowane jest za pomocą kwestionariuszy wyboru. Udział w badaniu jest nieobowiązkowy. Mają Państwo prawo wycofać się na każdym etapie badania. W przypadku rezygnacji wystarczy zamknąć okno przeglądarki, co spowoduje przerwanie procedury testowej, istniejące dane nie zostaną zapisane. Dane zostaną wysyłane dopiero po naciśnięciu okienka „wyślij" na końcu formularza i są równoznaczne ze świadomą zgodą na udział w badaniu.

Dziękujemy za poświęcony czas i zaangażowanie.

Płeć:

- kobieta
- mężczyzna

Wiek (w latach):

- ……

Obszar studiów:

- administracyjne
- artystyczne
- biologiczno-przyrodnicze
- ekonomiczne
- filologiczne

- humanistyczne
- medyczne
- pedagogiczne
- prawnicze
- psychologiczne
- rolnicze i leśnie
- ścisłe
- społeczne
- techniczne
- turystyczno-sportowe
- wojskowe i morskie

Czy obserwowałeś/aś u siebie objawy long COVID?

(Według aktualnej definicji zespół long COVID występuje u osób z potwierdzonym lub prawdopodobnym zakażeniem SARS-CoV-2, po 3 miesiącach od momentu zachorowania na COVID-19, u których objawy utrzymują się przynajmniej przez 2 kolejne miesiące lub dłużej. Charakterystyczne objawy dla long COVID to: objawy ogólne (zmęczenie lub zmęczenie utrudniające codzienne życie, objawy nasilające się po wysiłku fizycznym lub psychicznym, gorączka); objawy ze strony układu oddechowego i serca (trudności w oddychaniu lub duszność, kaszel, ból w klatce piersiowej, szybkie bicie lub kołatanie serca); objawy neurologiczne (trudności z myśleniem lub koncentracją, ból głowy, problemy ze snem, zawroty głowy podczas wstawania, uczucie mrowienia, zmiany węchu lub smaku, depresja lub stany lękowe); objawy ze strony układu pokarmowego (biegunka, ból brzucha); inne objawy (bóle stawów lub mięśni, wysypka, zmiany w cyklach menstruacyjnych).

- tak
- nie

…………………………………………………………………………………………..

Międzynarodowy Kwestionariusz Aktywności Fizycznej (IPAQ)

Prosimy teraz pomyśleć o wszystkich czynnościach wykonywanych w ciągu ostatnich 7 dni w domu i w jego otoczeniu, w pracy zawodowej, związanych z przemieszczaniem się z miejsca na miejsce, np. drodze do pracy i z pracy, robieniu zakupów. Proszę także uwzględnić czynności wykonywane w czasie wolnym, tj. spacery, rekreacja, praca na działce, ćwiczenia fizyczne oraz sport. Najpierw zapytamy Pana/Panią o czynności wymagające dużego wysiłku fizycznego, następnie o czynności wymagające umiarkowanego, średniego wysiłku, a na koniec o spacery i inne czynności związane z chodzeniem oraz siedzeniem.

1a. Czy w ciągu ostatnich 7 dni wykonywał/a Pan/Pani czynności wymagające intensywnego wysiłku fizycznego? Intensywnego wysiłku fizycznego wymaga np. dźwiganie ciężkich przedmiotów, kopanie ziemi, aerobik, szybki bieg, szybka jazda rowerem. Interesują nas tylko czynności, które trwały co najmniej 10 min. bez przerwy.

Tak – przez ile dni w ciągu ostatniego tygodnia? .... dni

Nie (przejść do pyt. 3)

Nie wiem/Nie jestem pewien(a) (przejść do pyt. 3)

1b. Przeciętnie ile czasu wykonywał/a P. czynności wymagające intensywnego wysiłku fizycznego w ciągu takiego dnia? .... minut dziennie Nie wiem/Nie jestem pewien(a)

Umiarkowanego wysiłku fizycznego wymaga np. noszenie lżejszych ciężarów, jazda rowerem w normalnym tempie, gra w siatkówkę lub bardzo szybki marsz. Proszę jednak nie brać pod uwagę chodzenia. Chodzi znowu tylko czynności, które trwały co najmniej 10 minut bez przerwy.

2a. Czy w ciągu ostatnich 7 dni wykonywał/a P. czynności wymagające umiarkowanego, średniego wysiłku fizycznego?

Tak – przez ile dni w ciągu ostatniego tygodnia? .... dni

Nie (przejść do pyt. 5)

Nie wiem/Nie jestem pewien(a) (przejść do pyt. 5)

2b. Przeciętnie ile czasu wykonywał/a P. czynności wymagające umiarkowanego wysiłku fizycznego w ciągu takiego dnia? .... minut dziennie

Nie wiem/Nie jestem pewien(a)

Teraz proszę przypomnieć sobie, ile czasu zajęło Panu/Pani chodzenie w ciągu ostatnich 7 dni. Interesuje nas chodzenie związane z pracą, chodzenie ulicą, np. po zakupy, do pracy, a także o spacery. Chodzi znowu o chodzenie, które trwało co najmniej 10 minut bez przerwy.

3a. Czy w ciągu ostatnich 7 dni chodził/a P. co najmniej 10 min. bez przerwy? Tak – przez ile dni w ciągu ostatniego tygodnia? .... dni

Nie (przejść do pyt. 7)

Nie wiem/Nie jestem pewien(a) (przejść do pyt. 7)

3b. Przeciętnie ile czasu poświęcał/a P. na chodzenie lub spacery w ciągu takiego dnia? .... minut dziennie

Nie wiem/Nie jestem pewien(a)

A ile czasu w ostatnim tygodniu spędzał Pan/Pani siedząc? Tym razem proszę uwzględnić tylko dni powszednie, tzn. proszę pominąć sobotę i niedzielę. Chodzi np. o siedzenie przy biurku, siedzenie podczas odwiedzin u znajomych, podczas czytania, a także siedzenie lub leżenie podczas oglądania telewizji. Proszę uwzględnić czas spędzony na siedzeniu w domu, w pracy, w szkole, w pojazdach i w innych miejscach.

4. Biorąc pod uwagę dni powszednie w ciągu ostatniego tygodnia, ile zazwyczaj czasu w ciągu dnia spędzał/a P. siedząc? .... minut dziennie

Nie wiem/Nie jestem pewien(a)

…………………………………………………………………………………………...

KWESTIONARIUSZ NIEPEŁNOSPRAWNOŚCI LĘDŹWIOWEJ (ODI)

Poniższy kwestionariusz pozwala na ocenę wpływu Pana/Pani dolegliwości **kręgosłupa lędźwiowego** na codzienne życie. W każdej sekcji należy zaznaczyć tylko JEDNĄ odpowiedź najtrafniej opisującą Pana/Pani stan z DZIŚ.

Nasilenie bólu

- W tej chwili nie odczuwam bólu.
- W tej chwili ból jest niewielki.
- W tej chwili ból jest średni.
- W tej chwili ból jest dość silny.
- W tej chwili ból jest bardzo silny.
- W tej chwili ból jest najgorszy, jaki mogę sobie wyobrazić.

Codzienne czynności (mycie, ubieranie, itp.)

- Mogę wykonywać samodzielnie codzienne czynności bez dodatkowego bólu.
- Mogę wykonywać samodzielnie codzienne czynności, ale wywołują one znaczny ból.
- Samodzielne wykonywanie codziennych czynności jest bardzo bolesne, wykonuję je powoli i uważnie.
- Wymagam pewnej pomocy przy wykonywaniu codziennych czynności, ale większość wykonuję samodzielnie.
- Potrzebuję pomocy każdego dnia przy wykonywaniu większości czynności.
- Nie jestem w stanie się ubrać, myję się z trudem, większość czasu spędzam w łóżku.

Chodzenie

- Ból wcale nie przeszkadza mi w chodzeniu.
- Ból nie pozwala mi chodzić dalej niż 1 – 2 km.
- Ból nie pozwala mi chodzić dalej niż 500 m.
- Ból nie pozwala mi chodzić dalej niż 100 m.
- Mogę chodzić jedynie o lasce lub o kulach.
- Pozostaję cały czas w łóżku, z wielkim trudem dostaję się do toalety.

Podnoszenie

- Mogę podnosić ciężkie przedmioty, bez dodatkowego bólu.
- Mogę podnosić ciężkie przedmioty, lecz powoduje to dodatkowy ból.
- Z powodu bólu nie mogę podnosić ciężkich przedmiotów z podłogi, lecz mogę podnosić je, gdy są dogodnie położone, na przykład na stole.
- Z powodu bólu nie mogę podnosić ciężkich przedmiotów, lecz mogę podnosić lekkie lub średnio ciężkie, gdy są dogodnie położone, na przykład na stole.
- Z powodu bólu mogę podnosić tylko bardzo lekkie przedmioty.
- Z powodu bólu nie mogę podnosić ani przenosić żadnych przedmiotów.

Siedzenie

- Mogę siedzieć na każdym krześle dowolną ilość czasu.
- Mogę siedzieć na moim ulubionym krześle dowolną ilość czasu.
- Z powodu bólu mogę siedzieć nie dłużej niż 1 godzinę.
- Z powodu bólu mogę siedzieć nie dłużej niż pół godziny.
- Z powodu bólu mogę siedzieć nie dłużej niż 10 minut.
- Z powodu bólu wcale nie mogę siedzieć.

Stanie

- Mogę stać dowolną ilość czasu bez dodatkowego bólu.
- Mogę stać dowolną ilość czasu, lecz powoduje to dodatkowy ból.
- Z powodu bólu nie mogę stać dłużej niż 1 godzinę.
- Z powodu bólu nie mogę stać dłużej niż 30 minut.
- Z powodu bólu nie mogę stać dłużej niż 10 minut.
- Z powodu bólu nie mogę w ogóle stać.

Spanie

- Mój sen nigdy nie jest zaburzony bólem.
- Mój sen czasem jest zaburzony bólem.
- Z powodu bólu nie śpię dłużej niż 6 godzin.
- Z powodu bólu nie śpię dłużej niż 4 godziny.
- Z powodu bólu nie śpię dłużej niż 2 godziny.

- Z powodu bólu w ogóle nie mogę spać.

Życie seksualne (jeśli dotyczy)

- Moje życie seksualne wygląda tak jak zwykle i nie wywołuje dodatkowego bólu.
- Moje życie seksualne wygląda tak jak zwykle, ale wywołuje dodatkowy ból.
- Moje życie seksualne wygląda w przybliżeniu tak jak zwykle, ale powoduje silny ból.
- Moje życie seksualne jest mocno ograniczone przez ból.
- Moje życie seksualne prawie nie istnieje z powodu bólu.
- Z powodu bólu w ogóle nie mogę prowadzić życia seksualnego.

Życie towarzyskie

- Moje życie towarzyskie jest normalne, nie powoduje dodatkowego bólu.
- Moje życie towarzyskie jest normalne, lecz powoduje dodatkowy ból.
- Ból nie ma znaczącego wpływu na moje życie towarzyskie, poza czynnościami związanymi z aktywnością fizyczną (np. sport).
- Ból ograniczył moje życie towarzyskie, z tego powodu częściej zostaję w domu.
- Ból ograniczył moje życie towarzyskie do spotkań w domu.
- Z powodu bólu nie prowadzę życia towarzyskiego.

Podróżowanie

- Mogę podróżować wszędzie bez bólu.
- Mogę podróżować wszędzie, lecz powoduje to dodatkowy ból.
- Ból jest silny, ale jestem w stanie podróżować ponad 2 godziny.
- Ból nie pozwala mi podróżować dłużej niż 1 godzinę.
- Ból ogranicza moje podróże do niezbędnych, trwających krócej niż 30 minut.
- Z powodu bólu podróżuję wyłącznie do miejsc związanych z leczeniem.

…………………………………………………………………………………………………...

WSKAŹNIK NIEPEŁNOSPRAWNOŚCI SZYJNEJ (NDI)

Poniższy kwestionariusz pozwala zrozumieć, w jaki sposób **ból szyi** oddziałuje na Pana/Pani możliwości wykonywania codziennych czynności. W każdej sekcji zaznaczy tylko JEDNĄ odpowiedź najbliżej opisującą Twój stan. Jeśli więcej niż jedna odpowiedź pasuje do Pana/Pani stanu, należy zaznaczyć w każdej sekcji jedną odpowiedź najlepiej oddającą Pana/Pani stan z dziś.

Nasilenie bólu

- W tej chwili nie odczuwam bólu.
- W tej chwili ból jest łagodny.
- Ból pojawia się i odchodzi i jest umiarkowany.
- Ból jest umiarkowany i w zasadzie stały.
- Ból jest silny, odchodzi i przychodzi.
- Ból jest silny i nie bardzo się zmienia.

Codzienne czynności (mycie, ubieranie, itp.)

- Mogę wykonywać samodzielnie codzienne czynności bez dodatkowego bólu.
- Mogę wykonywać samodzielnie codzienne czynności, ale wywołują one znaczny ból.
- Samodzielne wykonywanie codziennych czynności jest bardzo bolesne, wykonuję je powoli i uważnie.
- Wymagam pewnej pomocy przy wykonywaniu codziennych czynności, lecz większość wykonuję samodzielnie.
- Potrzebuję pomocy każdego dnia przy wykonywaniu większości czynności.
- Nie jestem w stanie się ubrać, myję się z trudem, większość czasu spędzam w łóżku

Podnoszenie

- Mogę podnosić ciężkie przedmioty bez dodatkowego bólu.
- Mogę podnosić ciężkie przedmioty, lecz powoduje to dodatkowy ból.
- Z powodu bólu nie mogę podnosić ciężkich przedmiotów z podłogi, lecz mogę je podnosić, gdy są dogodnie położone, na przykład na stole.
- Z powodu bólu nie mogę podnosić ciężkich przedmiotów, lecz mogę podnosić lekkie lub średnio ciężkie, gdy są dogodnie położone, na przykład na stole.
- Z powodu bólu mogę podnosić tylko bardzo lekkie przedmioty.
- Z powodu bólu nie mogę podnosić żadnych przedmiotów.

Czytanie

- Mogę czytać tyle, ile chcę nie odczuwając żadnego bólu karku.
- Mogę czytać tyle, ile chcę, lecz czytanie powoduje niewielki, dodatkowy ból karku.
- Mogę czytać tyle, ile chcę, lecz czytanie powoduje dodatkowy ból karku o średnim nasileniu.
- Muszę ograniczać czytanie z powodu dodatkowego bólu o średnim nasileniu.
- Prawie wcale nie mogę czytać z powodu silnego bólu.
- Z powodu bólu karku wcale nie jestem w stanie czytać.

Ból głowy

- Nie miewam bólów głowy.
- Ból głowy zdarza się rzadko i jest niewielki.
- Ból głowy zdarza się rzadko i ma średnie nasilenie.
- Często miewam bóle głowy o średnim nasileniu.
- Często miewam silne bóle głowy.
- Głowa boli mnie prawie cały czas.

Koncentracja

- Mogę się zawsze w pełni skoncentrować bez trudu.
- Mogę się zawsze w pełni skoncentrować, choć z niewielkimi trudnościami.
- Mam pewne trudności z koncentracją.
- Mam znaczne trudności z koncentracją.
- Mam bardzo duże trudności z koncentracją.
- W ogóle nie mogę się skoncentrować.

Praca

- Mogę pracować bez ograniczeń.
- Mogę wykonywać wyłącznie rutynowe czynności w pracy, lecz nie więcej.
- Mogę wykonywać większość moich obowiązkowych czynności w pracy.
- Nie jestem w stanie wykonywać rutynowych czynności w pracy.
- Z wielkim trudem wykonuję jakąkolwiek pracę.
- Z powodu bólu nie jestem w stanie pracować.

Jazda samochodem

- Mogę jeździć samochodem i nie powoduje to dodatkowego bólu karku.
- Mogę jeździć samochodem bez ograniczeń, lecz towarzyszy temu niewielki dodatkowy ból karku.
- Mogę jeździć samochodem bez ograniczeń, lecz towarzyszy temu dodatkowy bólu karku o średnim nasileniu.
- Nie mogę jeździć samochodem tyle, ile chcę z powodu bólu karku o średnim nasileniu.
- Prawie wcale nie jeżdżę samochodem z powodu silnego bólu karku.
- Wcale nie mogę jeździć samochodem z powodu bólu karku.

Spanie

- Ból w ogóle nie zaburza mojego snu.
- Ból zaburza mój sen w bardzo niewielkim stopniu (mniej niż 1 godzina bezsenności na dobę).
- Ból zaburza mój sen w niewielkim stopniu (1-2 godziny bezsenności na dobę).
- Ból zaburza mój sen w średnim stopniu (2-3 godziny bezsenności na dobę).

- Ból zaburza mój sen w znacznym stopniu (3-5 godzin bezsenności na dobę).
- Ból w ogóle nie pozwala mi spać (5-7 godzin bezsenności na dobę).

Rekreacja

- Mogę wykonywać wszystkie czynności rekreacyjne bez bólu.
- Mogę wykonywać wszystkie czynności rekreacyjne z niewielkim bólem karku.
- Z powodu bólu karku mogę wykonywać większość czynności rekreacyjnych – lecz nie wszystkie.
- Z powodu bólu karku nie jestem w stanie wykonywać większości typowych czynności rekreacyjnych.
- Mogę z trudem wykonywać tylko niektóre czynności rekreacyjne z powodu bólu karku.
- Z powodu bólu karku wcale nie wykonuję czynności rekreacyjnych.
